# Preparation Process Optimization of Peptides from *Agaricus blazei* Murrill, and Comparison of Their Antioxidant and Immune-Enhancing Activities Separated by Ultrafiltration Membrane Technology

**DOI:** 10.3390/foods12020251

**Published:** 2023-01-05

**Authors:** Xian-Guo Zou, Yun Chi, Yu-Qin Cao, Miao Zheng, Ze-Yuan Deng, Ming Cai, Kai Yang, Pei-Long Sun

**Affiliations:** 1 College of Food Science and Technology, Zhejiang University of Technology, Hangzhou 310014, China; 2 Key Laboratory of Food Macromolecular Resources Processing Technology Research, Zhejiang University of Technology, China National Light Industry, Hangzhou 310014, China; 3 State Key Laboratory of Food Science and Technology, Nanchang University, Nanchang 330047, China

**Keywords:** *Agaricus blazei*, ultrasound-assisted enzymatic extraction, ultrafiltration technology, peptide, antioxidant activity, immune-enhancing activity

## Abstract

*Agaricus blazei* murrill (ABM), a large fungus, is reported to have extensive biological activities but the antioxidant and immune-regulatory capacities have been less studied and the components responsible for the functions are unclear. This study prepared ABM peptides (ABMP) using ultrasound-assisted enzymatic extraction (UAEE) strategy and cascade ultrafiltration (UF) membrane technology. The UAEE extraction conditions were optimized using response surface methodology (RSM) with four factors and three levels to achieve the maximum ABMP yield (34.03%); the optimal conditions were an enzyme amount of 4%, ratio of ABM to water of 1:30, ultrasonic power of 360 W, and ultrasonic time of 30 min. Four ABMP fractions were obtained after UF with different pore size and their antioxidant and immune-regulatory abilities were evaluated and compared. The results showed that they could effectively scavenge DPPH, hydroxyl, and ABTS radicals, especially for ABMP-2; the scavenging rate of the above radicals were 79.31%, 63.60%, and 96.08%, respectively. In addition, four ABMP fractions also activated macrophage activity through strengthening phagocytosis and the production of NO, IL-6, IL-1β, and TNF-α in a dose-dependent manner. Notably, the ABMP-2 fraction with a MW of 3–5 kDa and peptide purity of 82.88% was found to have the best effect, showing the maximum phagocytosis (189.37%) as well as NO (7.98 μM), IL-6 (195.05 pg/mL), IL-1β (876.15 pg/mL), and TNF-α (1620 pg/mL) secretion at a treatment concentration of 150 μg/mL. The findings indicated that the ABMP, especially for the separate ABMP-2, could be used as dietary supplements and have the potential to be exploited as immune-enhancing agents.

## 1. Introduction

*Agaricus blazei* murrill (ABM) is a large fungus, belonging to the *Agaricus* (black umbrella) genus, and is commercially cultivated in Brazil (Cogumelo do Sol), China (Ji Song Rong), and Japan (Himematsutake) [1]. The fruiting body of ABM is rich in macronutrients including proteins (40–50%), polysaccharides (35–40%), and lipids (3–4%), and micronutrients, such as sterols and flavonoids, with a broad spectrum of biological properties, including anticancer, immunoregulatory, antioxidant, antihypertensive, and antiviral activities [2,3]. Fanhani [4] found that adding *Agaricus blazei* (0–0.2%) to the diet of broilers could provide a better immune response by stimulating immunoglobulins and macrophages, and the ABM compounds (possibly polyphenols) exerted antioxidant effects. Jiang [5] observed an ABM polysaccharide and found that it could enhance the body immunity by modulating the Th1 response. Wei [6] reported that the ABM extracts had in vitro antioxidant effects, but the active ingredient was unclear. Studies on the bioactivity of ABM peptides (ABMP) are limited. Souza [7] found that the partial hydrolysis of the ABM protein can inactivate free radicals and enhance the activity of the antioxidant enzymes, showing a significant antioxidant capacity, and had the advantages of stability and easy absorption. Nevertheless, the molecular weight (MW) range and purity of the partial hydrolysis were unknown.

Bioactive peptide preparation benefits from the increasing application of novel extraction technologies. The most common technique for producing bioactive peptides from parent protein molecules is through enzymatic hydrolysis since these reactions would not result in organic solvents residue or toxic chemicals in the final extracts [8]. Due to limited interaction between the enzyme and substrate, pretreatment methods such as ultrasonic-assisted extraction were preliminarily used to accelerate the extraction of parent protein molecules [9]. Yang [10] applied an ultrasound-assisted double enzyme hydrolysis and ultrafiltration approach for the low MW peptide extraction from bovine bone, showing better antioxidant activities. The selection of ultrasonic condition and enzyme amount is crucial as they are associated with the degree of hydrolysis, which can affect size and amino acid composition of peptide, and thus the bioactivities of the produced peptides [11].

Membrane separation technology, previously used in the chemical industry, has been applied to separate high-added-value compounds based on their size, and has the superiority of purity improvement, low cost, product safety, and mild processing conditions, in favor of maintaining the stability and bioactivity of valuable compounds to the full extent [12,13]. Ultrafiltration technology (UF) is the most used method of membrane-based processes for the purpose of separating bioactive components from natural products, reaching 56–100% separation efficiencies [14]. Sonklin [15] used UF to separate peptides from mung bean meal and found that the F4 fraction (MW less than 1 kDa) was more potent in scavenging free radicals of DPPH, hydroxyl, and superoxide, as well as improving metal chelating activity than other fractions. Moreover, Wen [16] used a divergent ultrasound-assisted enzymatic extraction (UAEE) as well as the UF method to prepare watermelon protein hydrolysates, showing that the hydrolysates with MW < 1 kDa had better antioxidant activity than other fractions with higher MW.

The immune system protects the body against foreign bacterial and viral infections or injury and reduces the incidence of diseases [17]. Hence, immunostimulation using biologically active natural compounds is an effective strategy to strengthen the body’s defense systems [18]. Macrophages are known to play a pivotal role in the immune defense and surveillance. Most natural bioactive compounds were reported to activate macrophages through improving phagocytic ability and secretion of NO and cytokines, which help to eliminate foreign materials [19].

Therefore, this study focused on the preparation of bioactive ABMP with immune-enhancing activity and the content of this work includes the following. (1) The extraction and separation of ABMP using the UAEE strategy and cascade UF technology with various pore sizes in which the extraction conditions were optimized by single factor experiment and response surface methodology (RSM). (2) The evaluation and comparison of antioxidant and immune-enhancing activities of different ABMP fractions.

## 2. Materials and Methods

### 2.1. Materials and Reagents

The ABM dried samples, AbML11 strain, were kindly provided by Zhejiang Huihe Biotechnology Co. (Lishui, China). BCA protein assay kit and NO one-step kit were purchased from Beyotime Biotechnology (Jiangsu, China). RAW264.7 cells were obtained from Chinese Academy of Sciences (Shanghai, China). Pepsin (3000 U/g protein), 3-(4,5-dimethylthiazol-2-yl)-2,5-diphenyltetrazolium bromide (MTT), and LPS (Escherichia coli 055: B5) were obtained from Sigma Chemical Co. (St. Louis, MO, USA). Penicillin-streptomycin antibodies (10 M), phosphate buffered saline (0.01 M, pH 7.2), fetal bovine serum (FBS), and Roswell Park Memorial Institute (RPMI-1640) were purchased from Invitrogen-Gibco (New York, NY, USA). ELISA kits (IL-6, IL-1β and TNF-α) were all purchased from Jiangsu Jingmei Biotechnology Co. (Yancheng, China). Other chemicals of analytical grade were purchased from Sinopharm Chemical Reagent Co., Ltd. (Shanghai, China).

### 2.2. Preparation of ABMP

The ABM powder was obtained by grounding the dried sample and filtrating through 80 mesh sieves. It was mixed with distilled water at 400 rpm, thoroughly homogenized, and then stirred for 24 h at 25 °C in a 500 mL beaker. The supernatant was collected after centrifugation (Qiu Zuo Instruments Co., Shanghai, China) and was further precipitated overnight with the addition of 85% saturated (NH_4_)_2_SO_4_ at RT. Thereafter, the precipitate was dissolved in distilled water for ultrasonic treatment, and was then hydrolyzed using the pepsin (3000 U/g) at a of pH 2.0 and temperature of 37 °C.

The extraction conditions including enzyme amount, ratio of ABM to water, ultrasonic powder, and ultrasonic time were optimized at the next step. Finally, the extract of ABMP was prepared after lyophilization.

### 2.3. Optimization of ABMP Extraction Conditions by Single-Factor Test and RSM

The ABMP were extracted by UAEE, and the following conditions were optimized: enzyme amount (1.5%, 2.5%, 3.5%, 4.5%, 5.5%), ratio of ABM to water (1:10, 1:20, 1:30, 1:40, 1:50), ultrasonic power (180, 240, 300, 360, 420 W), and ultrasonic time (10, 20, 30, 40, 50 min). Based on the results of single-factor experiments, the optimum extraction conditions for ABMP were estimated using independent and dependent variables by regression analysis and RSM analysis.

### 2.4. Ultrafiltration Experiment

The ABMP extract was dissolved in distilled water and subsequently passed through pre-washed ultrafiltration membranes of 10, 5, and 3 kDa. The ultrafiltration was processed in an ultrafiltration cup (Mosel Scientific Equipment Co., Shanghai, China) at the conditions of 0.2 MPa N_2_ pressure and RT. The various fractions (<3 kDa, 3–5 kDa, 5–10 kDa, >10 kDa) were separately collected and defined as ABMP-1, ABMP-2, ABMP-3, and ABMP-4, respectively. Samples of the above fractions were freeze-dried and stored at −20 °C. Peptide purity of each fraction was calculated as follows:

(1) $$\mathrm{Purity}\;\mathrm{of}\;\mathrm{peptide}\left(\%\right)=\frac{M_{2}}{M_{1}}\times 100$$

where M_1_ was the weight of each fraction; M_2_ was the content of peptide in each fraction determined by a BCA kit.

### 2.5. Determination of Antioxidant Properties

#### 2.5.1. DPPH Radical Scavenging Activity

The DPPH radical scavenging activity of different fractions was measured with reference to the method reported by Zhang [20] with a slight modification. In brief, different fractions of ABMP were dissolved with distilled water to the final concentration of 1 mg/mL. Then, the sample solutions (1 mL) were added with DPPH-95% ethanol (0.1 mM, 1 mL). The mixture was left to stand for 30 min under dark at RT, and the absorbance (A_S_) was measured at 517 nm using the microplate reader (Biotek Synergy H1, BioTek Instruments Inc., California, USA). The samples included ABMP-1, ABMP-2, ABMP-3, and ABMP-4, and vitamin C (VC) was a positive control. Each fraction was replicated thrice and determined five times. The value was detected as follows:

(2) $$\mathrm{DPPH}\;\mathrm{radical}\;\mathrm{scavenging}\;\left(\%\right)=\left[\frac{A_{0}-A_{s}}{A_{0}}\right]\times 100$$

where A_s_ is the absorbance value of each sample or VC; A_0_ is the absorbance value of 95% ethanol.

#### 2.5.2. Hydroxyl Radical Scavenging Activity

The antioxidant activity of the ABMP was also evaluated by the detection of their ability to clear hydroxyl radicals using the method described by Zhang [21]. An aliquot (1 mL) of ABMP samples (1 mg/mL), FeSO_4_ (6 mmol/L), and H_2_O_2_ (6 mmol/L) solutions were added to a centrifuge tube and mixed. After mixing, we added 1 mL salicylic acid-anhydrous ethanol (6 mmol/L) to the former centrifuge tube, and then the mixture was reacted for 30 min at 37 °C in a water bath; the absorbance of the final reaction mixture was measured at 510 nm (A_H_) by a microplate reader. In the control group, the ABMP sample was replaced by deionized water. The samples included ABMP-1, ABMP-2, ABMP-3, and ABMP-4, and VC was a positive control. Each fraction was replicated thrice and determined five times The value of the hydroxyl radical scavenging ability was calculated as follows:

(3) $$\mathrm{Hydroxyl}\;\mathrm{radical}\;\mathrm{scavenging}\;\left(\%\right)=\left[1-\frac{A_{0}-A_{H}}{A_{I}}\right]\times 100$$

where A_H_ is the absorbance value of each sample or VC; A_I_ is the absorbance value of the sample without salicylic acid; A_0_ is the absorbance value of deionized water.

#### 2.5.3. ABTS Radical Scavenging Activity

The determination of ABTS clearance was measured using the method described by Zhu [22]. Certain quantities of ABTS were dissolved in deionized water and the ABTS solution at a concentration of 7.4 mM was obtained. The K_2_S_2_O_8_ solution of 2.6 mM was also prepared after dissolving the potassium persulfate in deionized water. The ABTS free radical solution was prepared with the mixing of ABTS and K_2_S_2_O_8_ solutions (stored for 12 h under dark) and was diluted to give an absorbance of 0.7 at 735 nm before use. The ABTS radical solution was mixed with different fractions of ABMP solution (1 mg/mL) at a ratio of 20:1 and reacted at RT for 6 min. At the end of the reaction, the absorbance value was measured at 735 nm (A_c_) by a microplate reader. In the control group, the ABMP sample was replaced by deionized water. The samples included ABMP-1, ABMP-2, ABMP-3, and ABMP-4, and VC was a positive control. Each fraction was replicated thrice and determined five times. The ABTS radical scavenging ability of each sample was determined as follows:

(4) $$\mathrm{ABTS}\;\mathrm{radical}\;\mathrm{scavenging}\;\left(\%\right)=\left[\frac{A_{0}-A_{C}}{A_{0}}\right]\times 100$$

where A_c_ is the absorbance value of each sample or VC; A_0_ is the absorbance value of the diluted ABTS stock solution.

### 2.6. Determination of Cell Viabilities

The RAW264.7 mouse macrophages were cultured in RPMI 1640 medium supplemented with 10% FBS and 1% penicillin-streptomycin at 37 °C in a humidified atmosphere with 5% CO_2_. RAW264.7 cells in the logarithmic growth period were adjusted to 1 × 10^4^ cells/mL and inoculated in 96-well plates at 200 μL per well for 12 h. Thereafter, the culture medium was removed, and the cells were treated with 200 μL of different concentrations of ABMP fractions (0, 10, 50, 100, 150, 200 μg/mL) or LPS (1 μg/mL, positive control) for 24 h. Subsequently, 20 μL of 5 mg/mL MTT reagent was added to each well and incubated for an additional 4 h at 37 °C in the dark. After discarding the supernatant, 150 μL of DMSO solution was added to dissolve the formazan crystals, and the absorbance of each well was measured at OD 490 nm by a microplate reader. Cell viability of each sample was calculated using the following equation:

(5) $$\mathrm{Cell}\;\mathrm{viabilities}\;\left(\%\right)=\frac{A_{v}}{A_{0}}\times 100$$

where A_v_ is the absorbance value of each sample or LPS group; A_0_ is the absorbance value of the control group (without any treatment).

### 2.7. Determination of Cell Phagocytosis

The neutral red uptake assay was used to measure the effects of ABMP fractions on the phagocytic activity [23]. Briefly, RAW 264.7 cells (1 × 10^4^ cells/well) were seeded into 96-well plates overnight, and then incubated with various ABMP fractions (0, 50, 100, 150 μg/mL) or LPS for 24 h. Thereafter, neutral red solution of 100 µL (0.075%, dissolved in PBS) was added to the cells and incubated for another 1 h. After washing three times with PBS, the neutral red in cells was fully dissolved by the addition of lysis buffer (ethanol:glacial acetic acid = 1:1) for 4 h. The absorbance at OD 540 nm was measured by a microplate reader, and the phagocytosis rate of each sample was calculated using the following equation:

(6) $$\mathrm{Phagocytosis}\;\mathrm{rate}\;\left(\%\right)=\frac{A_{G}}{A_{0}}\times 100$$

where A_G_ is the absorbance value of each sample or LPS group; A_0_ is the absorbance value of the control group (without any treatment).

### 2.8. Determination of NO and Cytokine Secretion

The production of NO and cytokines were determined referring to the method described by Wu [19]. RAW264.7 cells (2.5 × 10^5^ cells/mL, 2 mL) were cultured in 6-well plates, and then administered with various concentrations of ABMP fractions (0, 50, 100, 150 μg/mL) and LPS, respectively, for 24 h. After collecting the supernatants to a new 96-well plate, Greiss reagent was added and incubated for 15 min. The absorbance was measured at 540 nm, which was used to calculate NO concentration by the usage of a standard curve obtained using sodium nitrate. Additionally, the secretion of cytokines (TNF-α, IL-6, and IL-1β) were examined by corresponding ELISA kits according to the manufacturer’s instructions.

### 2.9. Statistical Analysis

All tests were conduct in triplicate to minimize deviation, and data were presented as mean ± SD. Design-Expert 12.0 was applied to perform response surface analysis. RSM with a four-variable-three-level Box-Behnken design (BBD) was used to optimize the UAEE process. The four independent variables and three levels were: enzyme amount (2.5%, 3.5%, 4.5%), ratio of ABM to water (1:20, 1:30, 1:40), ultrasonic power (240, 300, 360 W), and ultrasonic time (20, 30, 40 min), respectively, and ABMP yield was the response value. The differences among mean values were evaluated using one-way ANOVA and Turkey HSD test with the help of the SPSS 17.0 software. A value of *p* < 0.05 (*), *p* < 0.01 (**), and *p* < 0.001 (***) indicated statistical significance.

## 3. Results and Discussion

### 3.1. Single Factor Experiment Analysis of UAEE

#### 3.1.1. Effect of Enzyme Amount on the ABMP Yield

**Figure 1:**
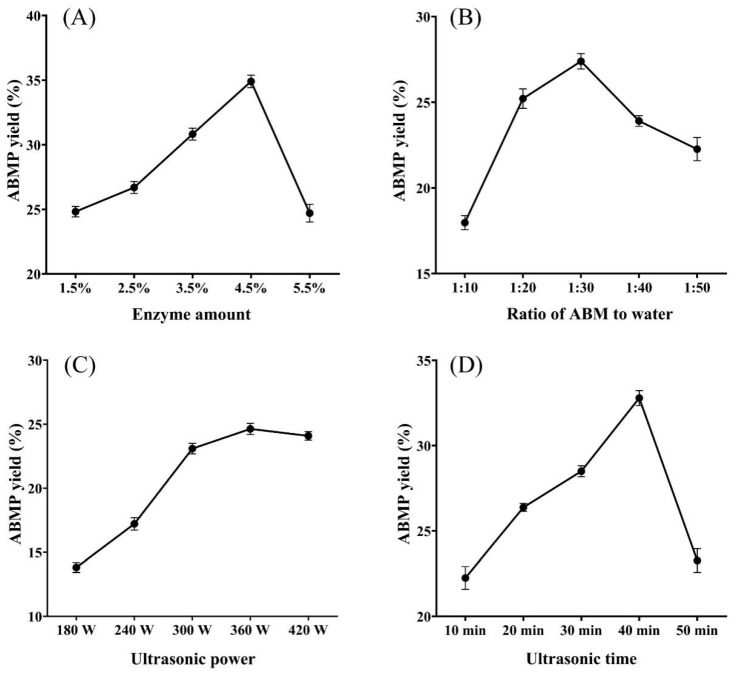
Effect of different extraction factors on the ABMP yield. (**A**) Enzyme amount; (**B**) ratio of ABM to water; (**C**) ultrasonic power; (**D**) ultrasonic time.

The amount of enzymes can influence the peptide yield. As shown in Figure 1A, with the increase of enzyme amount from 1.5% to 4.5%, the ABMP yield significantly elevated from 24.82% to 34.90%. However, when the enzyme dose further increased to 5.5%, the ABMP yield decreased to 24.70%, instead of increasing. This was possibly due to the excessive enzymes causing the peptides to be further hydrolyzed into amino acids, thus resulting in a decreased ABMP yield [24]. The results suggested that the enzyme amount of 4.5% was sufficient to obtain a high ABMP yield.

#### 3.1.2. Effect of the Ratio of ABM to Water on the ABMP Yield

The yield of ABMP extracted by different ratios of ABM to water from 1:10 to 1:50 is presented in Figure 1B. The ABMP yield increased from 17.97% to 27.39% with the ratio increasing from 1:10 to 1:30. When the ratio further increased (1:40), the ABMP yield significantly reduced. This phenomenon might be because the high ratio of water probably reduced the collision between substrate and enzyme, and thus weakened the enzymatic hydrolysis reaction [25]. Therefore, an extraction ratio of 1:30 was favorable for ABMP production. Cao [26] also found when the water/material ratio was 30.0, the hydrolysis degree of edible bird’s nest reached the highest (9.43 ± 0.30%).

#### 3.1.3. Effect of Ultrasonic Power on the ABMP Yield

As UAEE was used to extract ABMP, ultrasonic power was another important factor affecting the peptide yield. As shown in Figure 1C, the ABMP yield elevated from 13.81% to 24.63% with the increase of ultrasonic power from 180 W to 360 W, which was slightly decreased at an ultrasonic power of 420 W. Subhedar [27] reported that appropriate ultrasonic power was conducive to breaking up cells, leading to the release of proteins/peptides. Instead, excessive ultrasonic power would destroy the solubilized proteins/peptides, resulting in reduced yield [28]. Similar to our study, the solubility and peptide yield of peanut proteins reported by Li et al. were significantly improved after appropriate ultrasonic treatment [29].

#### 3.1.4. Effect of Ultrasonic Time on the ABMP Yield

The ultrasonic effect not only depends on the ultrasonic powder but also relies on the ultrasonic time. As demonstrated in Figure 1D, the shorter ultrasonic time, the lower peptide yielded when the ultrasonic time was between 10–40 min. However, excessive ultrasonic time (50 min) would lead to decreased ABMP yield, possibly because the prolonged ultrasonic time resulted in excessive temperature and energy [30], destroying the peptides’ structure. Therefore, the optimum ABMP yield was 32.79% at an ultrasonic time of 40 min.

According to the single factor results, the following conditions were adopted for RSM analysis: enzyme amount of 2.5–4.5% (*w*/*w*); ratio of ABM to water of 1:20 to 1:40; ultrasonic power of 300–420 W; and ultrasonic time of 20–40 min.

### 3.2. Response Surface Analysis of UAEE

#### 3.2.1. Predicted Mathematical Model and Statistical Analysis

Box-Behnken design (BBD) with four factors and three levels were performed to optimize the interactive effects of four independent variables (enzyme amount, ratio of ABM to water, ultrasonic power, and ultrasonic time) on the ABMP yield. The design matrix and the ABMP yield are shown in Table 1. The experimental data were further analyzed by multiple regression analysis, and the predicted model reflecting the relationship between response variable and the independent variable was presented as the following second-order polynomial equation:

Y (%) = 29.33 + 3.77A + 1.49B − 0.78C − 1.96D + 1.31AB + 1.45AC + 2.76AD + 1.21BC + 2.55BD − 0.49CD − 3.18A^2^ − 5.36B^2^ − 3.92C^2^ − 1.60D^2^ (7)

where Y represents the dependent variable of ABMP yield; A, B, C, and D represent the factors of enzyme amount, ratio of ABM to water, ultrasonic power, and ultrasonic time, respectively. AB, AC, AD, BC, BD, and CD represent the interaction of enzyme amount and ratio of ABM to water, enzyme amount and ultrasonic power, enzyme amount and ultrasonic time, ratio of ABM to water and ultrasonic power, ratio of ABM to water and ultrasonic time, and ultrasonic power and ultrasonic time, respectively.

The rational analysis of regression model including variance, goodness-of-fit, and the adequacy are summarized in Table 2. The *F*-value and *p*-value of the model were 7.19 and 0.0004, respectively, suggesting that the model was statistically significant. Furthermore, the lack of fit of the above two values was 1.16 and 0.4811, confirming the goodness-of-fit and suitability of the regression model. The high adjusted determination coefficient (R_Adj_^2^ = 0.7558) and low coefficient variation value (C.V.% = 8.15%) further revealed that the experimental values of the regression model were precise and reliable. The results in Table 2 also exhibited that the linear coefficients (A and D), interaction coefficients (AD and BD), and quadratic term coefficients (A^2^, B^2^ and C^2^) were significant (*p* < 0.05) while the other term coefficients were insignificant (*p* > 0.05).

#### 3.2.2. Response Surface Plot and Contour Plot

**Figure 2:**
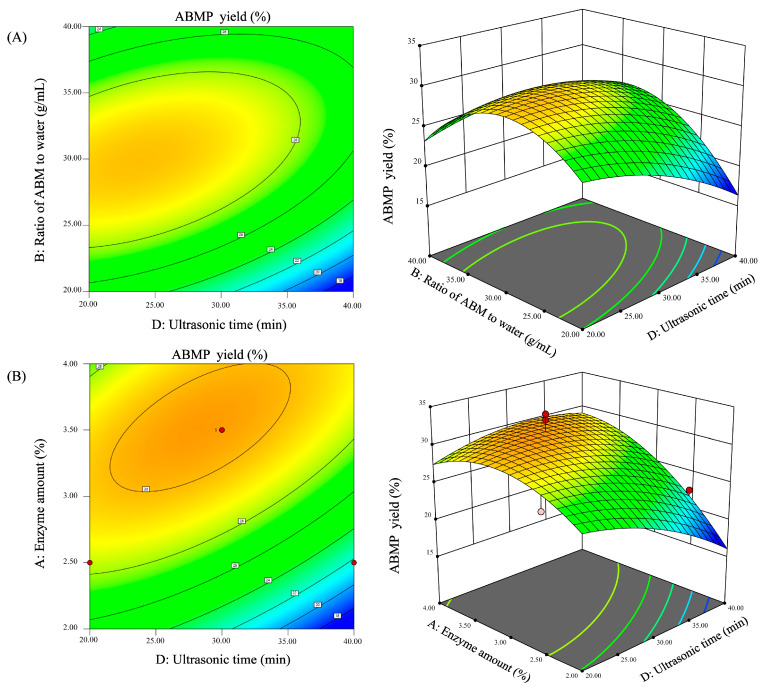
Response surface and contour map of interactive effect between different factors. (**A**) Interaction between ultrasonic time and ratio of ABM to water. (**B**) Interaction between ultrasonic time and enzyme amount.

3D response surface and 2D contour plots can graphically represent the regression function, showing a visualization of the mutual effects between two tested variables and the relationship between response value and different levels of variables. In the present study, the mutual effect of test factors on the ABMP yield is listed in Figure 2. It can be seen that the mutual interactions between ratio of ABM to water and ultrasonic time were significant. In addition, enzyme concentration and ultrasonic time both imposed significant effects on the ABMP yield.

#### 3.2.3. Verification of Predictive Model

By analyzing the plots in Figure 2, the optimal UAEE conditions for yielding 29.80% ABMP were as follows: enzyme amount of 3.86, ratio of ABM to water of 1:33.69, ultrasonic power of 365.10 W, and ultrasonic time of 33.68 min. To confirm the reliability and accuracy of the predicted results, the experiment was carried out under slightly modified optimal conditions: enzyme amount of 4%, ratio of ABM to water of 1:30, ultrasonic power of 360 W, and ultrasonic time of 30 min. The experimental yield of ABMP was 34.03%, which was higher than the theoretical predicted results. Therefore, the RSM model was suitable for optimizing the conditions of ABMP production.

### 3.3. Effect of Ultrafiltration on the Peptide Purity of ABMP

The peptide content of ABMP prepared under optimal conditions was 38.75%, which was improved using an ultrafiltration membrane of different pore sizes (10, 5, and 3 kDa). As presented in Table 3, four fractions named ABMP-1 (<3 kDa), ABMP-2 (3–5 kDa), ABMP-3 (5–10 kDa), and ABMP-4 (>10 kDa) were obtained, and their peptide purity was 35.38%, 84.87%, 82.88%, and 24.14%, respectively. It is obvious that ABMP-2 and ABMP-3 had high peptide purity. Although the weight of ABMP-4 (MW > 10 kDa) among all fractions was the highest (688.5 mg), it had the lowest peptide purity. The reason could be that other macromolecules, such as polysaccharides, might be present in this fraction. Furthermore, the low peptide purity of the ABMP-1 fraction was possibly due to the existence of some flavonoids, polyphenols, and others.

### 3.4. Effect of Different Fractions of ABMP on the Antioxidant Activity

**Figure 3:**
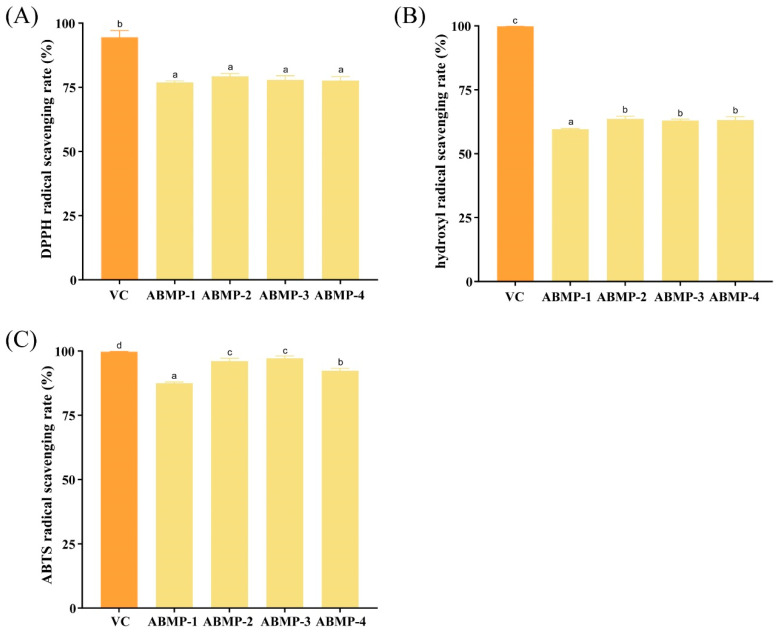
Antioxidant capacity of different fractions of ABMP. (**A**) DPPH radical scavenging rate; (**B**) Hydroxyl; (**C**) ABTS radical scavenging rate. Results were expressed as means ± SD (n = 3). Statistical significance analysis between different groups was done by the Tukey HSD test. Values marked with different letters (a, b, c, and d) were significantly different (*p* < 0.05). VC was a positive control group.

The antioxidant activities of different fractions of ABMP (1 mg/mL) were compared by evaluating their abilities to scavenge DPPH, hydroxyl, and ABTS radicals. The DPPH radical scavenging rates of ABMP-1, ABMP-2, ABMP-3, and ABMP-4 were 76.90%, 79.31%, 77.93%, and 77.59%, respectively, showing no significant differences (*p* > 0.05) (Figure 3A). This phenomenon might be because other compounds in ABMP-1 and ABMP-4 could bind to DPPH radicals. The hydroxyl radical scavenging capacities of the four fractions were not significantly different and were lower than that of VC (99.87%, 1 mg/mL) (Figure 3B), suggesting the poor ability of ABMPs to react with hydroxyl radicals. It was reported that hydroxyl radicals could be scavenged by decomposing with aromatic compounds into phenoxy groups or generating peroxy groups [31]. However, the aromatic amino acids in peptides from *Agaricus blazei* are lesser, making it difficult to scavenge hydroxyl radicals [1]. Compared with the other fractions, ABMP-2 (96.08%) and ABMP-3 (97.18%) were similar and had the highest ABTS radical scavenging capacity; the effect was close to the positive VC group (Figure 3C). Overall, ABMP-2 and ABMP-3 at the MW range of 3–10 kDa had the optimum free radical scavenging abilities, possibly due to their high purity of peptides. The results in our study were different from a previous work, showing that DPPH radical scavenging capacity of peptides from *Agaricus bisporus* was in the following order: 1–3 kDa > 3–5 kDa > 5–10 kDa [32]. This might be because the different extraction method (acidolysis extraction) resulted in large differences in amino acid composition among different fractions [33,34], or because the peptide purity with MW of 1–3 kDa in their study was higher than ours, although they did not detect the purity.

### 3.5. Effect of Different Fractions of ABMP on the Immunoregulatory Capability of RAW264.7 Cells

#### 3.5.1. Effect of Different Fractions of ABMP on Cell Viabilities

**Figure 4:**
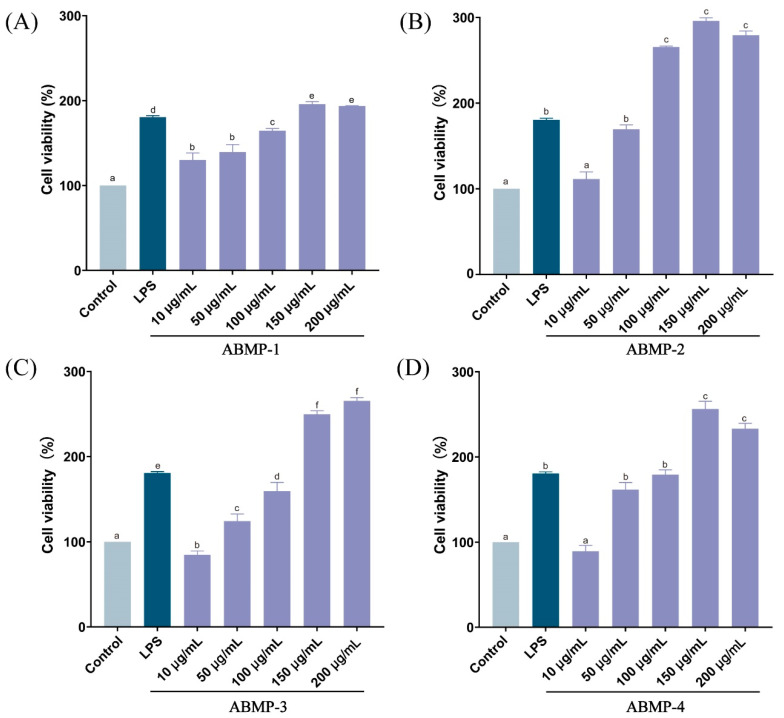
Effects of different fractions of ABMP on RAW264.7 cell viabilities. (**A**) ABMP-1; (**B**) ABMP-2; (**C**) ABMP-3; (**D**) ABMP-4. Mean values marked with different letters (a, b, c, d, etc.) were significantly different (*p* < 0.05).

Macrophage activation plays a pivotal role in strengthening the body’s innate and adaptive immune defense capabilities [19]. Herein, the immune-regulatory activities of four ABMP fractions (ABMP-1, ABMP-2, ABMP-3, and ABMP-4) were investigated using RAW264.7 murine macrophages. The viability of RAW264.7 cells administered with four ABMP fractions at different concentrations (10, 50, 100, 150, and 200 µg/mL) were examined and compared. As shown in Figure 4, all ABMP fractions at a concentration range of 50–200 µg/mL increased cell proliferation instead of exhibiting cytotoxic effects, and cell viability differed significantly among different concentrations. When the concentrations of ABMP-2 and ABMP-4 increased to 200 µg/mL, the cell viabilities slightly reduced compared with the lower concentration. Therefore, ABMP fractions at the concentrations of 50–150 µg/mL were used for the following study in which concentrations of the cell viability of each fraction significantly increased in a dose-dependent manner compared with the control group.

#### 3.5.2. Effect of Different Fractions of ABMP on Macrophage Phagocytosis

**Figure 5:**
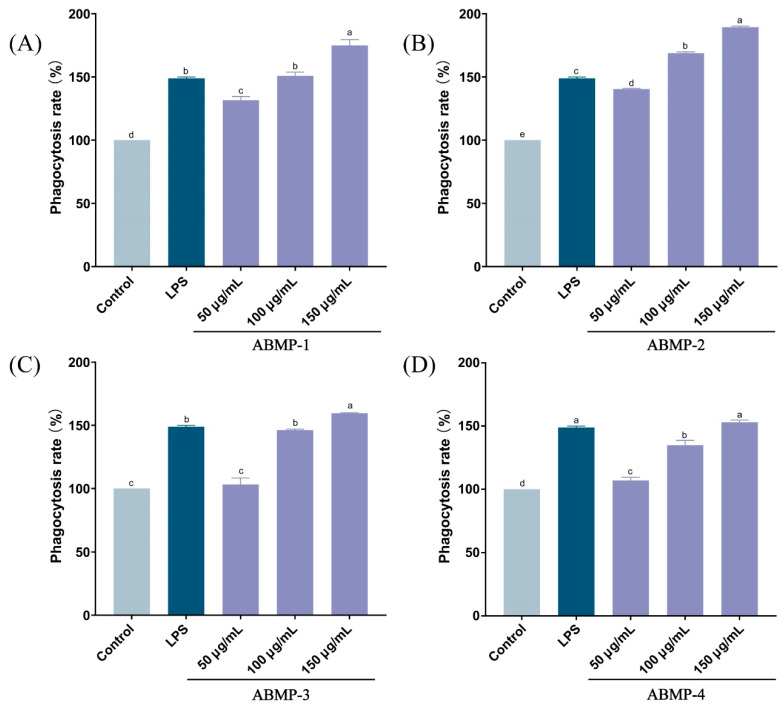
Effects of different fractions of ABMP on RAW264.7 phagocytosis rates. (**A**) ABMP-1; (**B**) ABMP-2; (**C**) ABMP-3; (**D**) ABMP-4. Mean values marked with different letters (a, b, c, d, etc.) were significantly different (*p* < 0.05).

Once activated by pathogenic organisms or external stimulus, macrophages can exert a variety of biological functions for defense, such as phagocytosis, production of chemotactic cytokines, and destruction of targeted organisms [35]. To explore whether ABMP fractions could activate macrophages, we tested the phagocytic ability using the neutral red uptake assay. As shown in Figure 5, the phagocytic capacity of RAW264.7 cells treated with all fractions (50–150 μg/mL) was found significantly increased (*p* < 0.05) in a concentration-dependent manner. In particular, compared with other fractions, ABMP-2 had higher phagocytic activity. For example, the phagocytosis rate of ABMP-2 treated at the highest concentration was 189.37%, which was about twice as high as the control group and about 50% higher than the positive control LPS (1 μg/mL). The results showed that all ABMP fractions promoted the phagocytosis function of RAW264.7 cells and the best effect was observed for ABMP-2, which might be due to its lower MW and higher peptide purity (Table 3). Supporting our speculation, Wang [36] concluded that low MW peptides had better structural stability and absorption. Zhang [37] found that peptides with lower MW (3–10 kDa) from the *Sporisorium reilianum* displayed a higher phagocytosis rate than the fraction of MW > 10 kDa.

#### 3.5.3. Effect of Different Fractions of ABMP on NO and Cytokines Secretions

**Figure 6:**
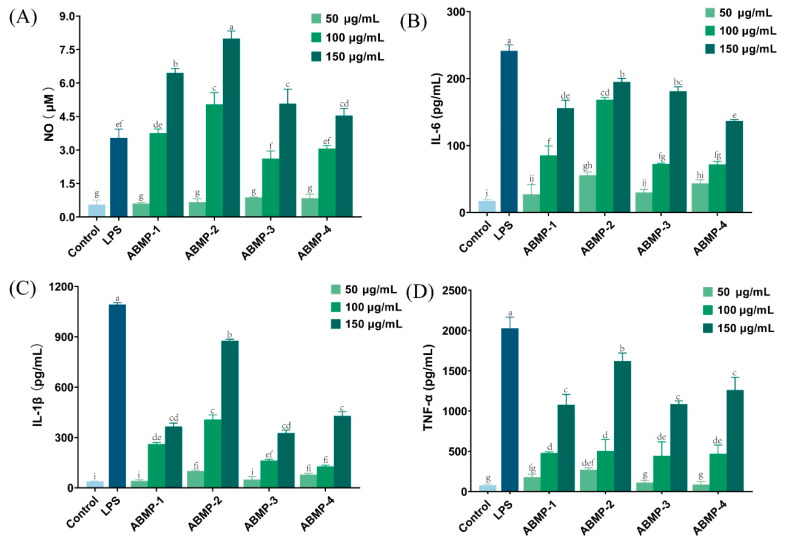
Effects of different fractions of ABMP on the production of NO and cytokines. (**A**) NO; (**B**) IL-6; (**C**) IL-1β; (**D**) TNF-α. Mean values marked with different letters (a, b, c, d, etc.) were significantly different (*p* < 0.05).

NO is an important effector molecule produced by the immune system, which is involved in host immune defense and is lethal to intracellular parasites and bacteria [38]. In this study, all ABMP fractions stimulated macrophages to produce NO in a dose-dependent manner (Figure 6). In particular, the NO production of ABMP-1, ABMP-2, ABMP-3, and ABMP-4 at a high dose (150 μg/mL) was 6.45, 7.98, 5.07, and 4.53 μM, respectively, higher than that of the positive control group. The higher NO secretion in ABMP-2 was consistent with the phagocytosis result, suggesting that the immune peptide sequences in ABMP-2 might be high. Cai [39] found that a *Thunnas albacares* peptide (550–1300 Da) had the same immunomodulatory effect and showed the same dose-dependent relationship of NO secretion in RAW264.7 cells.

In addition to NO, macrophages secrete inflammatory factors such as IL-6, IL-1β, and TNF-α, which are also thought to promote cellular immune function [40]. IL-1β and IL-6 can enhance the body’s immune response by facilitating the differentiation of T and B lymphocytes [41,42]. TNF-α can enhance the immune response by clearing harmful pathogens and causing the apoptosis of tumor cells [43]. Therefore, in this study, the levels of IL-6, IL-1β, and TNF-α were measured using ELISA kits to further compare the immune-regulatory activity of different ABMP fractions. All ABMP fractions had similar dose-dependent manner effects on the IL-6, IL-1β, and TNF-α production in RAW264.7 cells (Figure 6B–D). It is worth noting that the generation of the above three cytokines treated by ABMP-2 was the highest among all fractions, and the levels were detected as 195.05, 876.15, and 1620 pg/mL, respectively, at a treatment concentration of 150 μg/mL. In addition, the reinforcement of ABMP-2 on the cytokine production were found to all be significantly lower than those of LPS, indicating that ABMP-2 exerted moderate immune-enhancing activity. Similar to the present results, Sun [44] investigated the immune-modulatory activity of peptides from *Agaricus mycelium*, finding that they also promoted IL-6 and TNF-α secretion of RAW264.7 macrophages in a dose-dependent manner. Taken together, ABMP-2 with lower MW and high purity had the strongest immune-enhancing activity.

## 4. Conclusions

In summary, four ABMP fractions with different MW range and purity were prepared using the UAEE extraction strategy and cascade UF separation technology under the optimal extraction conditions. After evaluating the capacity to scavenge DPPH, hydroxyl, and ABTS free radicals, all four ABMP fractions were found to effectively eliminate the aforementioned radicals, especially for ABMP-2 and ABMP-3. In addition, they were found to enhance the phagocytic ability and improve the generation of NO and cytokines IL-6, IL-1β, and TNF-α in RAW264.7 macrophages, and the effect was potent for ABMP-2 with low MW and high purity. These results suggest that ABMP fractions, especially for ABMP-2, displayed excellent antioxidant and immune-enhancing activities, thus can be used as dietary supplements or exploited as antioxidants and immune modulators. Future studies will concentrate on elucidating the peptide composition and structure of ABMP-2 fractions.

## Figures and Tables

**Table 1:** Box-Behnken design matrix and response values for the ABMP yield.

	Four Factors with Three Levels				Response Values
Sequence	Enzyme Amount (A, %)	Ratio of ABM to Water (B, g/mL)	Ultrasonic Power (C, W)	Ultrasonic Time (D, min)	ABMP Yield (%)
1	3.5	1:30	360	30	32.79
2	3.5	1:40	360	40	27.28
3	3.5	1:20	360	40	17.49
4	3.5	1:30	360	30	29.71
5	3.5	1:30	360	30	31.99
6	2.5	1:30	360	40	22.48
7	3.5	1:20	300	30	20.46
8	4.5	1:30	420	30	23.89
9	4.5	1:30	360	20	24.77
10	4.5	1:30	360	40	31.92
11	3.5	1:30	420	20	25.77
12	3.5	1:30	420	40	21.11
13	3.5	1:40	360	20	23.62
14	2.5	1:30	300	30	24.93
15	3.5	1:30	300	20	26.89
16	4.5	1:30	300	30	20.86
17	3.5	1:20	360	20	24.03
18	2.5	1:30	360	20	26.38
19	3.5	1:20	420	30	19.67
20	4.5	1:20	360	30	18.92
21	2.5	1:40	360	30	22.04
22	3.5	1:40	420	30	25.74
23	3.5	1:30	360	30	29.31
24	4.5	1:40	360	30	25.42
25	2.5	1:30	420	30	22.17
26	3.5	1:40	300	30	21.70
27	3.5	1:30	360	30	28.27
28	3.5	1:30	300	40	24.19
29	2.5	1:20	360	30	20.13

**Table 2:** Analysis of the variance (ANOVA) for regression model.

Source	Sum of Sqares	df	Mean Square	*F*-Value	*p*-Value	Significance
Model	405.07	14	28.93	7.19	0.0004	***
A-Enzyme amount	59.74	1	59.74	14.85	0.0018	**
B-Ratio of ABM to water	15.26	1	15.26	3.79	0.0718	
C-Ultrasonic power	4.18	1	4.18	1.04	0.3256	
D-Ultrasonic time	26.44	1	26.44	6.57	0.0225	*
AB	6.81	1	6.81	1.69	0.2142	
AC	8.38	1	8.38	2.08	0.1709	
AD	30.5	1	30.5	7.59	0.0155	*
BC	5.83	1	5.83	1.45	0.2485	
BD	26.01	1	26.01	6.46	0.0235	*
CD	0.96	1	0.96	0.24	0.6327	
BC	5.83	1	5.83	1.45	0.2485	
A^2^	65.66	1	65.66	16.32	0.0012	**
B^2^	186.2	1	186.2	46.28	<0.0001	***
C^2^	99.63	1	99.63	24.76	0.0002	***
D^2^	16.6	1	16.6	4.13	0.0616	
Lack of Fit	41.88	10	4.19	1.16	0.4811	
Pure Error	14.44	4	3.61			
Cor Total	461.39	28				
R^2^ = 0.8779		R_Adj_^2^ = 0.7558			C. V.% = 8.15%	

* *p* < 0.05, ** *p* < 0.01, *** *p* < 0.001.

**Table 3:** Total weight and peptide purity of different ABMP fractions.

ABMP Fractions	Total Weight (mg)	Content of Peptide (mg)	Purity of Peptide
Total peptides before ultrafiltration	1000	387.52	38.75%
ABMP-1 (<3 kDa)	69.3	24.52	35.38%
ABMP-2 (3–5 kDa)	101.7	84.29	82.88%
ABMP-3 (5–10 kDa)	126	106.94	84.87%
ABMP-4 (>10 kDa)	688.5	166.185	24.14%

## Data Availability

Data is contained within the article.
